# Supplementary material for: Two-qubit logic and teleportation with mobile spin qubits in silicon
Source: Nature. 2026 May 6;653(8114):391–7. doi: 10.1038/s41586-026-10423-9 (PMC13171600; doi:10.1038/s41586-026-10423-9)
Supplement: Supplementary file 1 — Supplementary Notes A–G, including Supplementary Figs. 1–11 and Supplementary Tables 1–4. [file 41586_2026_10423_MOESM1_ESM.pdf]

nature portfolio

https://doi.org/10.1038/s41586-026-10423-9

Supplementary information

Two-qubit logic and teleportation with mobile spin qubits in silicon

In the format provided by the
authors and unedited

This supplementary information includes:

- Supplementary Note A Device fabrication
- Supplementary Note B The concept and advantages of a mobile spin qubit architecture
- Supplementary Note C Potential simulation methodology
- Supplementary Note D Simulation of the tunnel coupling between moving quantum dots in the two conveyors
- Supplementary Note E Quantitative analysis of error source in CZ gate operation
- Supplementary Note F Characterization of polarization and phase information teleportation
- Supplementary Note G Quantitative analysis of error sources in quantum state teleportation

SUPPLEMENTARY INFORMATION

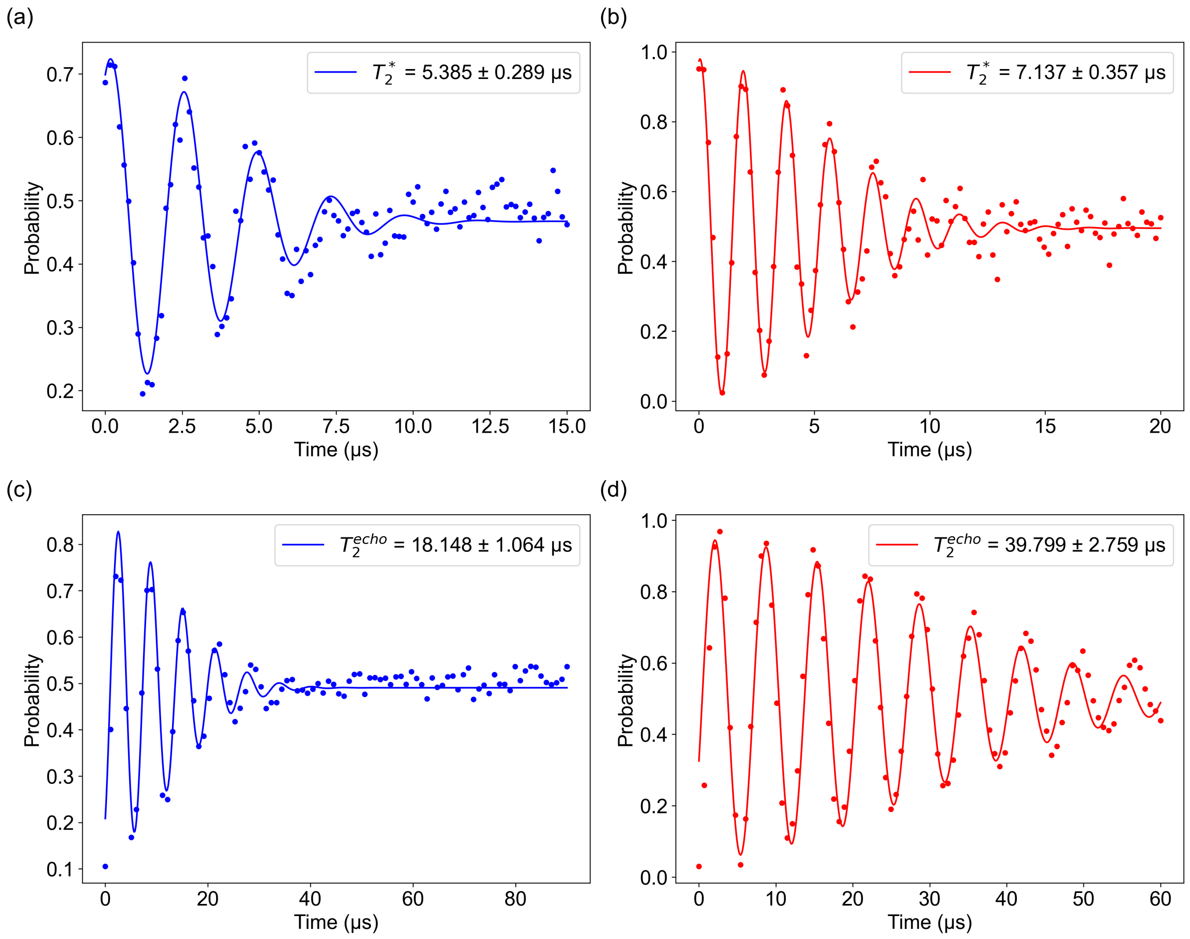

Supplementary Figure 1. **Ramsey and Echo measurement of Q2 and Q5** a,b) Ramsey interference measurements showing free induction decay for Q2 and Q5, yielding dephasing times T_2^* of 5.385 ± 0.289 μs and 7.137 ± 0.357 μs, respectively. The solid lines represent fits to the data using Gaussian-decayed sinusoidal functions of the form $A\exp(-(t/T_2^*)^2)\sin(2\pi f t + \phi) + C$. c,d) Hahn echo measurements for Q2 and Q5, demonstrating extended coherence times T_2^{echo} of 18.146 ± 1.064 μs and 39.799 ± 2.759 μs, respectively. The solid lines show the Gaussian-decayed sinusoidal fits to the echo data. The error bars represent one standard deviation extracted from the fit.

Supplementary Data Table I. Two-electron singlet–triplet energy splitting E_{ST} for each quantum dot, measured via magnetospectroscopy in [1] under a different cooldown and gate configuration from the present work. In strongly confined dots, E_{ST} provides a lower bound for the single-particle valley splitting E_v.

QD 1	QD 2	QD 3	QD 4	QD 5	QD 6
208 μeV	174 μeV	276 μeV	208 μeV	243 μeV	278 μeV

A. Device fabrication

In this work, the device is fabricated on a ^{28}Si/SiGe heterostructure [4]. Initially, a 1.5 μm thick, linearly graded $Si_{1-x}Ge_x$ buffer layer is deposited on a silicon wafer, which is then capped with a 300 nm-thick relaxed $Si_{0.7}Ge_{0.3}$ spacer. Next, a 7 nm-thick, tensile-strained ^{28}Si quantum well enriched to 800 ppm is grown [1]. A 30 nm thick $Si_{0.7}Ge_{0.3}$ spacer, passivated with dichlorosilane at 500°C [5], is employed to separate the quantum well from the gate stack. Ohmic contacts to the two-dimensional electron gas within the quantum well are then formed via phosphorus ion implantation. Following this, a 10 nm Al_2O_3 layer is deposited,

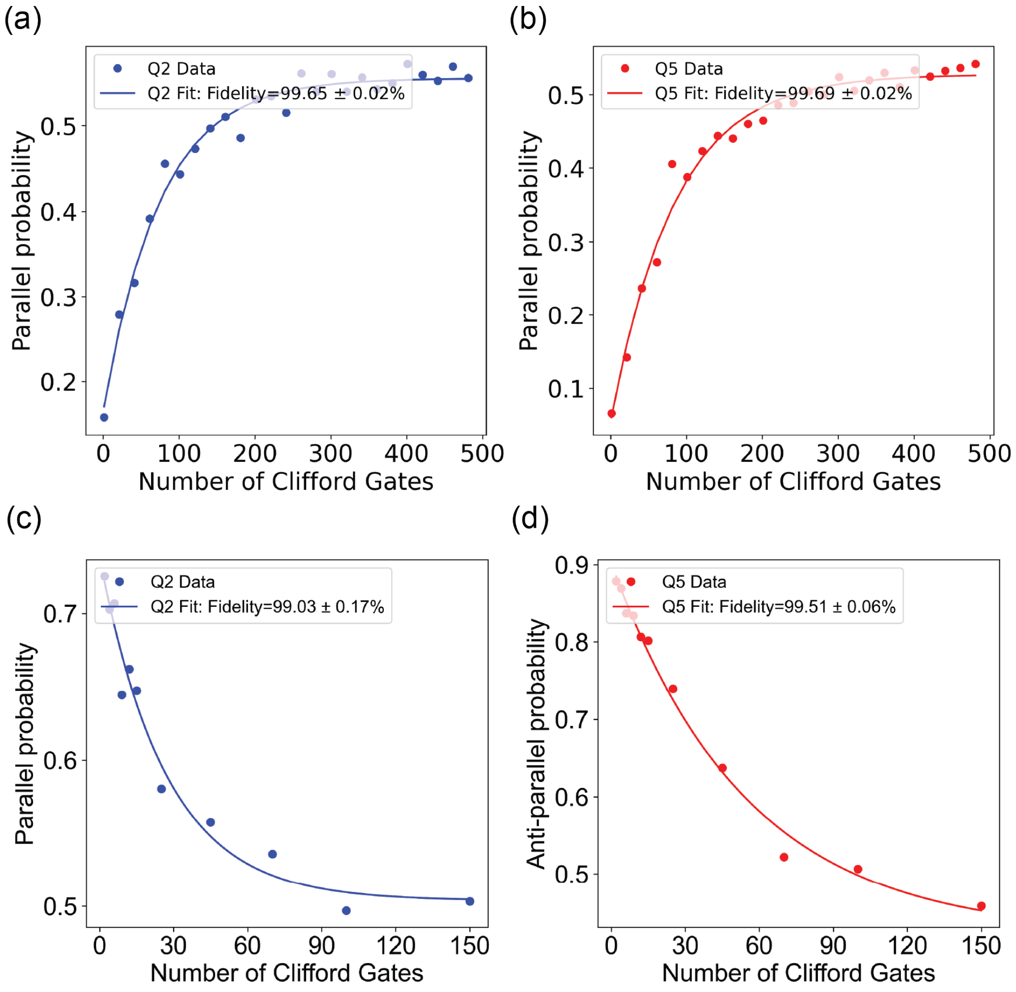

Supplementary Figure 2. **Randomized benchmarking of Q2 and Q5** a) Randomized benchmarking data for Q2 showing the parallel spin probability as a function of the number of Clifford gates. The solid line shows a fit yielding an average single-qubit gate fidelity of 99.65 ± 0.02%. b) Same measurement for Q5 yielding an average single-qubit gate fidelity of 99.69 ± 0.02%. c) Randomized benchmarking with simultaneous driving of Q2 and Q5, showing reduced fidelity for Q2 at 99.03 ± 0.17%. The degradation is likely due to heating-induced frequency shifts of the qubit [2]. d) Simultaneous driving measurement for Q5 showing fidelity of 99.51 ± 0.06%, which exhibits less degradation compared to Q2. The overall joint fidelity for simultaneous operation is 98.54 ± 0.18%. The error bars represent one standard deviation extracted from the fit.

succeeded by three Ti:Pd layers (with thicknesses of 3:17, 3:27, and 3:37 nm) deposited by electron beam evaporation; each metallic layer is interleaved with a 5 nm Al_2O_3 film grown by atomic layer deposition. Finally, an extra 5 nm Al_2O_3 layer is added atop the gate stack, upon which a 5:200 nm Ti:Co micromagnet is deposited to enable qubit control via magnetic field gradients.

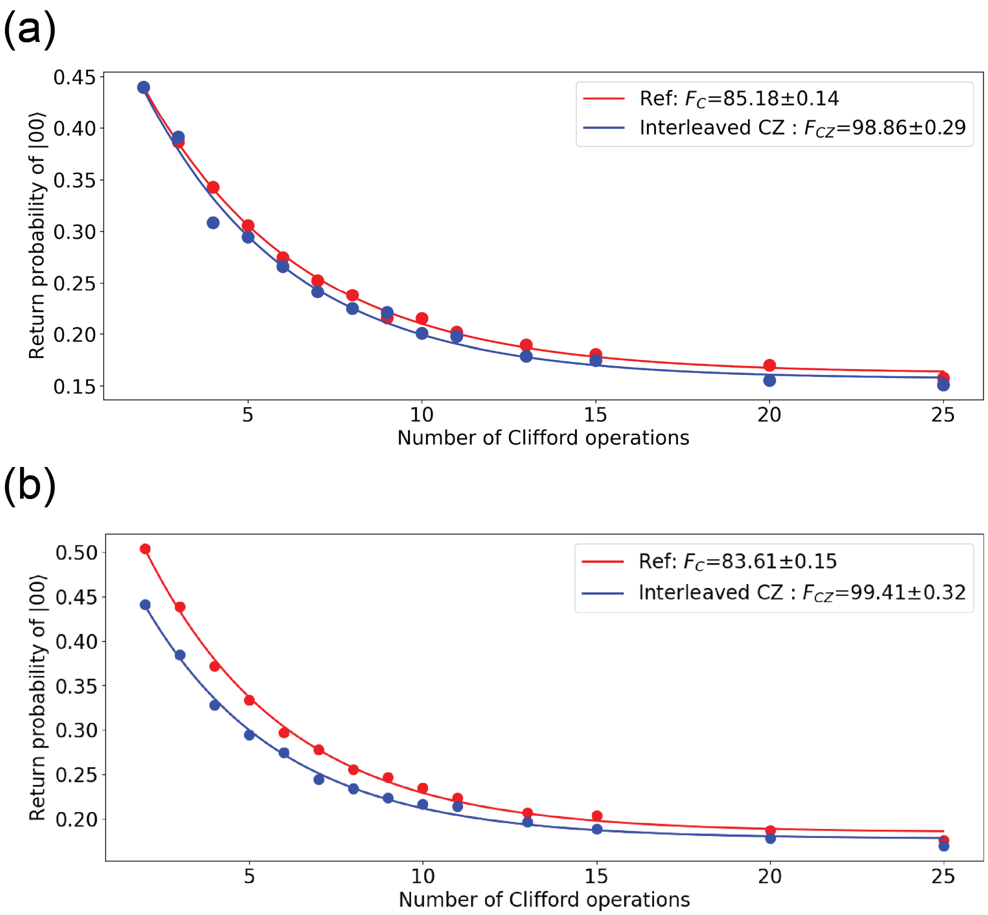

Supplementary Figure 3. **Multiple interleaved randomized benchmarking measurements.** a) First set of measurements showing sequence fidelity versus number of Clifford operations for both reference (red) and interleaved CZ gate (blue) sequences. The measured average Clifford gate fidelity is $F_C = 85.18 \pm 0.14\%$ and the extracted CZ gate fidelity is $F_{CZ} = 98.86 \pm 0.29\%$. b) Second set of measurements showing sequence fidelity versus number of Clifford operations for both reference (red) and interleaved CZ gate (blue) sequences, yielding an average Clifford gate fidelity of $F_C = 83.61 \pm 0.15\%$ and a CZ gate fidelity of $F_{CZ} = 99.41 \pm 0.32\%$. This dataset is not included in the main manuscript due to the significant amplitude difference between the two decay curves, which likely results from low-frequency drift affecting single-qubit gate errors and state preparation and measurement (SPAM) errors over the course of the measurement. The error bars represent one standard deviation extracted from bootstrap resampling.

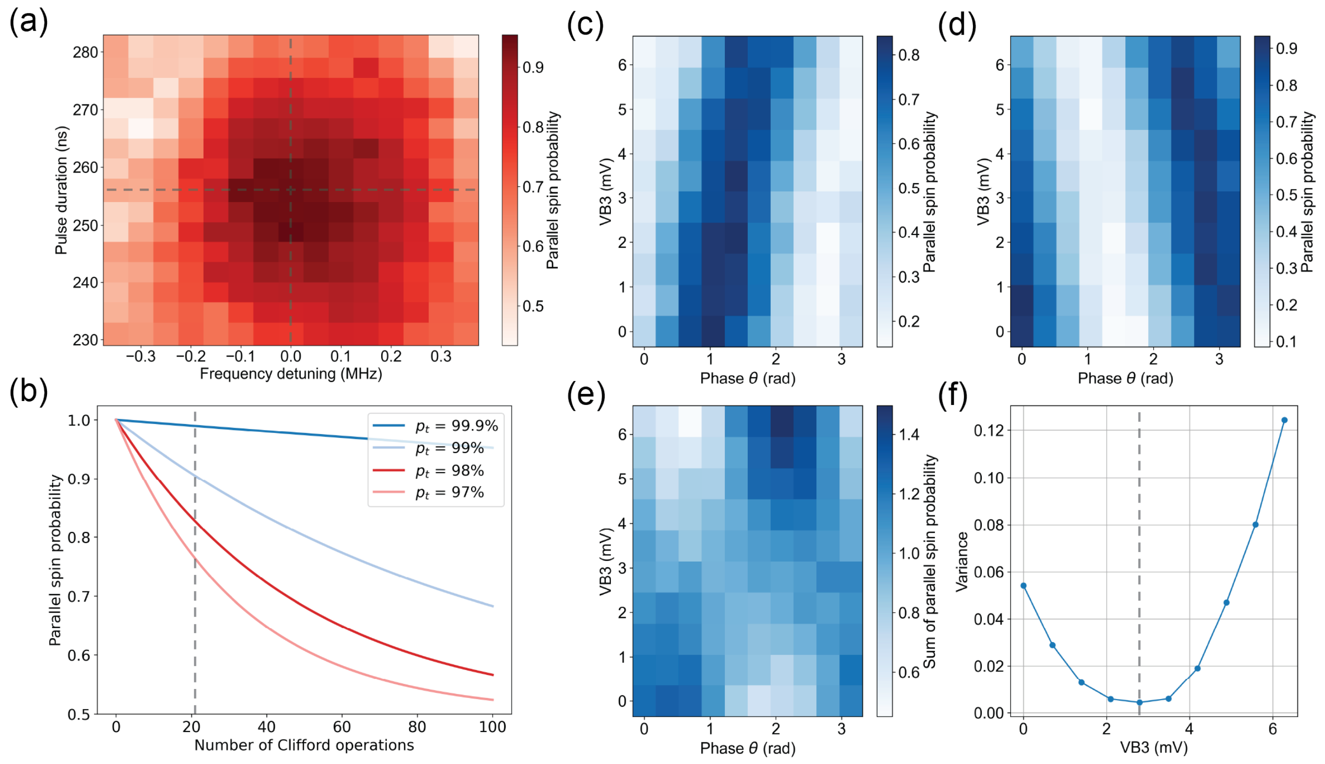

Supplementary Figure 4. **Single- and two-qubit gate optimization.** a) Two-dimensional scan of single-qubit gate calibration for Q5, showing the sequence fidelity of the RB with 20 Clifford gates as a function of microwave frequency detuning and $R_x(\pi/2)$ rotation pulse duration. The initial state is prepared in the excited state to avoid false optimization at fully off-resonant conditions. The dashed lines indicate the calibrated value of parameters. Similar optimization procedures were also performed for the CZ gate after the initial tuning shown in panels (c-f). b) shows the simulated parallel spin probability as a function of the number of Clifford gates in a RB sequence under different depolarizing factors p_t. It demonstrates why maximizing the parallel spin probability for a fixed number of Clifford gates leads to improved fidelity. c,d) Controlled-phase rotation angle of Q5 as a function of barrier gate B3 pulsed offset, with control qubit Q2 prepared in $|0\rangle$ and $|1\rangle$ states respectively, for a total gate time of 54 ns. e) Combined data from (c) and (d), where the optimal CZ gate condition corresponds to a phase evolution that is independent of the B3 voltage (horizontal stripe pattern). f) Variance of the phase evolution along the x-axis in (e). The minimum indicates the optimal B3 pulsed offset for implementing the CZ gate at the given gate duration [3]. The solid line only connects the datapoint and is a guide to the eye.

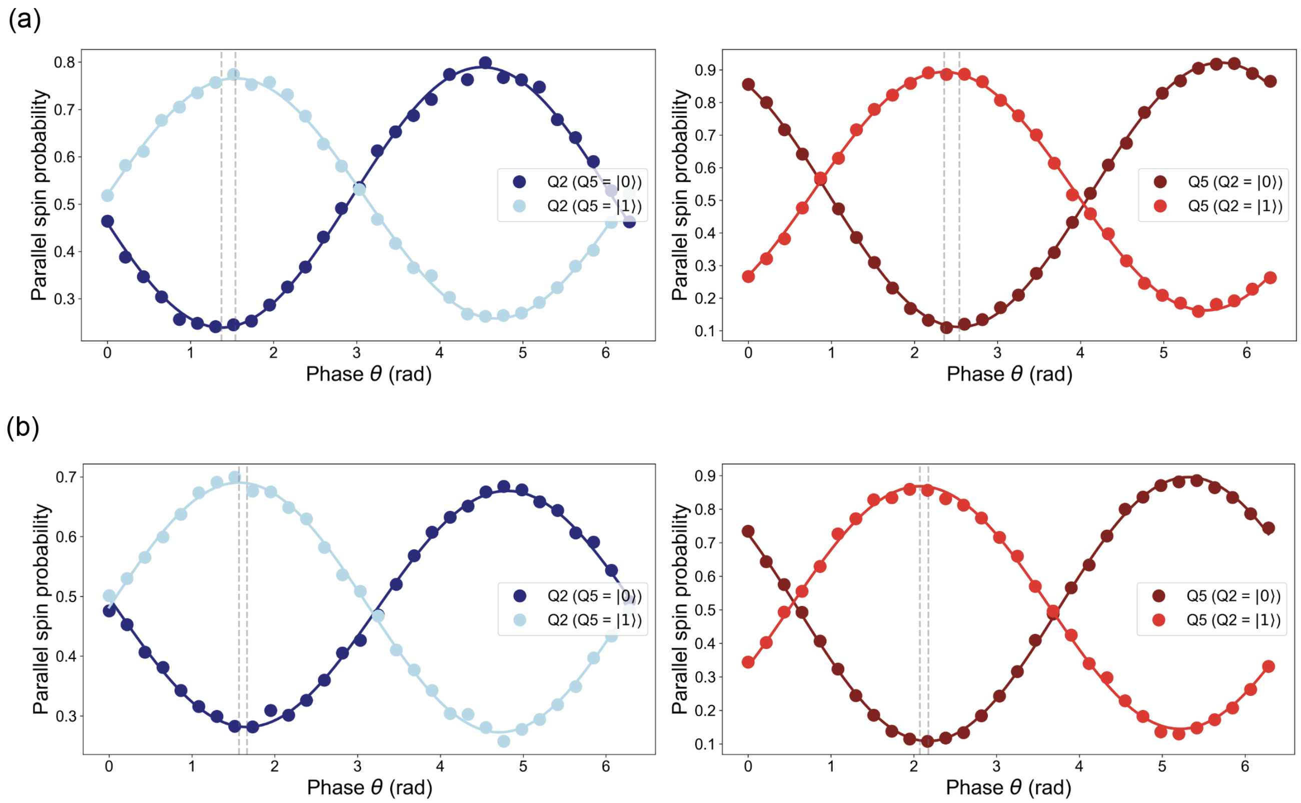

Supplementary Figure 5. **Heating effect on CZ calibration** Heating effect on CZ calibration. a) CZ calibration scan without MW pre-burst. b) CZ calibration scan with an off-resonant 8 μs MW pre-burst applied before qubit initialization. For both panels, after initialization, we measure the parallel spin probability using a sequence where one spin (Q2 for left panel, Q5 for right panel) is prepared in a superposition state using an Rx(π/2) gate while the other spin is initialized in either $|0\rangle$ or $|1\rangle$. The presence of the MW pre-burst significantly alters the phase evolution during the shuttling based CZ operation, illustrating how MW-induced heating can affect calibration accuracy. This effect may be caused by MW-induced shifts in qubit frequencies, which impact the phase acquisition during the operation [2]. This effect, rather than imperfect CZ operations, explains the slight deviation from a π controlled phase observed in the main text Figure 3c.

Supplementary Data Table II. Conveyor mode control parameters for Fig. 1e and Fig. 2b. The phase offsets exceed 2π to explicitly represent the moving directions.

Gate	Amplitude (mV)	Pulsed Offset (mV)	Phase Offset ϕ/θ (2π)
VP1	0	0	-0.7/0.0
VB1	0	-80	0.0/0.05
VP2	120	-90	0.1/0.3
B2	120	140	0.6/0.55
P3	120	-120	1.1/0.8
B3	100	V_{B3}	1.6/1.05
P4	120	85	1.1/0.8
B4	120	140	0.6/0.55
P5	120	-120	0.1/0.3
B5	0	-90	0.0/0.0
P6	0	50	0.0/0.0
B6	0	-30	0.0/0.0

Supplementary Data Table III. Conveyor mode control parameters for high fidelity CZ operations (Fig. 2(d) and Fig. 3. Here the voltage on P3 and P4 are finely tuned to improve the charge symmetry between Q2 and Q5. This configuration is used for Fig. 2d, Fig. 3, and Fig. 4.

Gate	Amplitude (mV)	Pulsed Offset (mV)	Phase Offset ϕ/θ (2π)
VP1	0	0	-0.7/0.0
VB1	0	-80	0.0/0.05
VP2	120	-90	0.1/0.3
B2	120	140	0.6/0.55
P3	120	-110	1.1/0.8
B3	100	9.5	1.6/1.05
P4	120	95	1.1/0.8
B4	120	140	0.6/0.55
P5	120	-120	0.1/0.3
B5	0	-90	0.0/0.0
P6	0	50	0.0/0.0
B6	0	-30	0.0/0.0

Supplementary Data Table IV. Conveyor mode control parameters for merging two elongated moving potentials. This configuration is used for Fig. 2f

Gate	Amplitude (mV)	Pulsed Offset (mV)	Phase Offset θ (2π)
VP1	0	0	0.0
VB1	0	-80	0.05
VP2	160	-90	0.3
B2	160	140	0.55
P3	160	-110	0.8
B3	160	-10	1.05
P4	160	80	0.8
B4	160	140	0.55
P5	160	-120	0.3
B5	0	-90	0.0
P6	0	50	0.0
B6	0	-30	0.0

B. The concept and advantages of a mobile spin qubit architecture

This section describes a mobile qubit architecture based on two shared-control conveyor belts and a central barrier gate, highlighting its advantages over conventional methods. Figures 6a,b illustrate two possible geometries derived from this concept.

Throughout the main text, we employ two-tone conveyor operation, where two phase-shifted sinusoidal signals are applied to ensure that nearest-neighbor potential minima are not unintentionally hybridized during shuttling. However, the fundamental principle of conveyor-mode shuttling does not inherently require two tones. Similar isolation of the moving potential minima

can also be achieved with a single-tone approach by optimizing the gate pitch at the device-design level [6], increasing the amplitude of the sinusoidal drive, or reducing static potential disorder. Since this section focuses on the conceptual discussion of architecture and scalability, we describe the conveyor operation in terms of a single-tone scheme for simplicity. The extension to two-tone operation is straightforward and does not alter the architectural considerations discussed here.

Fig.6a depicts an unit cell of the “shelf" architecture where two conveyor belts and a central barrier are arranged serially. The primary advantage of this configuration is the enhanced connectivity it provides between sparsely distributed qubit storage zones. The number of control electrodes per unit cell required for this scheme scales with the number of storage zones N as 4 (for one conveyor) $\times$ 2 + 1 (for the barrier between the conveyors) + $2N$ (for the storage dots).

A key operational characteristic is shown in Figure 6c, which plots the potential energy landscape at different phases of the conveyor cycle. The potentials generated by the two conveyors collide at the central barrier, which prevents qubits from directly passing each other. This is a drawback compared to the parallel geometry. However, as shown by the connectivity graph in Fig. 6e, this architecture can in principle achieve all-to-all connectivity among qubits connected to the same conveyor row. This is made possible by operating the central barrier as a dynamic extension of the conveyors, allowing a qubit to be shuttled from the left side to the right so that also spins originally located on the same side of the central barrier can be brought together to interact.

A practical limitation of the serial (shelf) configuration is that when a spin reaches the interaction zone, the conveyor operation must be locally halted or its effective transport direction reversed to allow the qubits to remain within the interaction region for the duration of the two-qubit gate. As a consequence, the number of qubits that can be simultaneously transported on a single conveyor belt and coupled within the same interaction zone is limited, making the achievable degree of parallelism for two-qubit gates a potential bottleneck. One possible mitigation strategy is to implement two-qubit gates between a conveyor channel and a nearby storage zone. These could be either SWAP-type gates or CPhase-type gates, the latter requiring nanomagnets in proximity to the storage regions, at the cost of enhanced decoherence. Therefore the optimal architecture depends on a nontrivial balance between gate time, two-qubit gate fidelity, and coherence during idling and transport.

In contrast, the “parallel" geometry shown in Fig. 6b features a unit cell including two conveyor belts arranged in parallel, separated by a horizontal barrier gate. As simulated in Fig. 6d, this configuration allows qubits to be brought together and interact via the horizontal barrier and subsequently pass each other, enabling all-to-all connectivity between qubits hosted in the parallel channels.

The most significant advantage of the parallel geometry is its scalability in terms of control hardware. The number of electrodes per unit cell is constant ($4 \times 2 + 1 = 9$) independent of the number of qubits. In this scheme, the practical limit on the number of qubits is determined by the maximum distance over which high-fidelity shuttling can be maintained in the conveyors.

The main trade-off compared to the shelf architecture is the loss of direct connectivity between qubits located on the same conveyor. However, in the implementation of common quantum error correction protocols such as the surface code, data and ancilla qubits can be segregated onto separate conveyors and interactions between data and ancilla qubits are sufficient. In this case, the parallel architecture meets the required connectivity [7] (as depicted in Fig. 6f). Fig. 7 shows how the unit cells from Fig. 6 can be assembled and connected together in an extended two-dimensional lay-out.

Next to the scaling of the number of electrodes needed for control, another important consideration for scalability is the number of control signals required to control all the conveyors. In the most general case, each unit cell would require an independent set of phase-shifted sinusoidal signals, implying that the number of digital-to-analog converter (DAC) channels scales with the number of unit cells. However, the effective DAC count can be substantially reduced through two complementary strategies. First, specialized cryogenic and room-temperature electronics, such as multi-phase signal generators or delay-line-based phase shifters [8], allow a single DAC to generate multiple phase-shifted outputs. Second, transversal gate operations in fault-tolerant quantum computing impose strong structure on control patterns: by optimizing the spatial assignment of logical qubits and restricting operations to those needed for transversal gates, control signals could possibly be shared across multiple conveyor belts, enabling global or semi-global broadcasting while only a limited subset of control lines remains locally tunable for calibration and error mitigation.

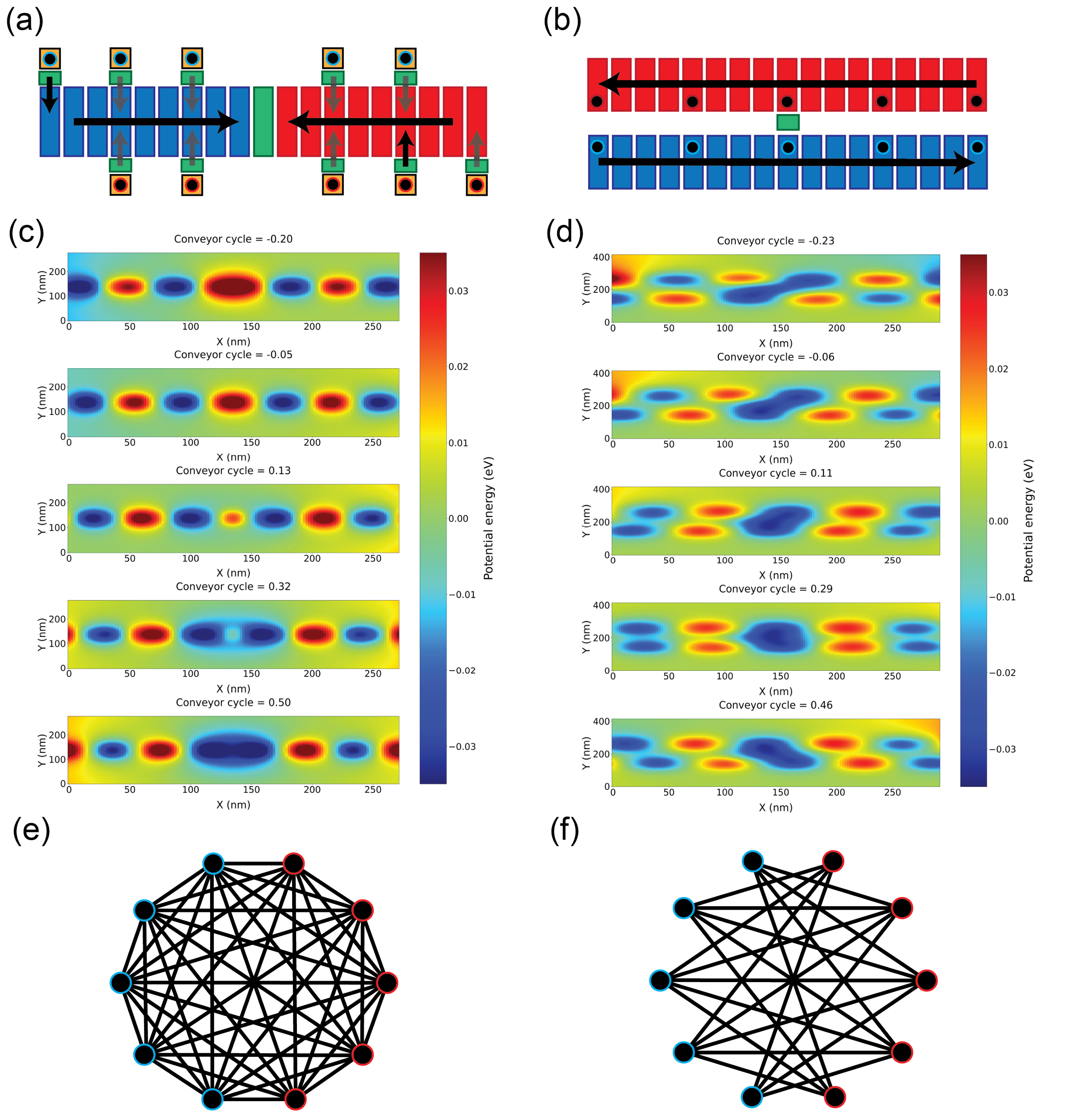

Supplementary Figure 6. **Conveyor-based mobile qubit architectures** (a) Shelf architecture. The red and blue gate arrays function as independent shared-control conveyor belts, while the green gate denotes an individual barrier gate. Red and blue circles indicate qubits stored (shelved) in the lower and upper storage zones, respectively. The arrows indicate possible shuttling paths, with their transparency illustrating shared-control constraints. Darker arrows show actively occupied paths in this example, while lighter arrows represent alternative paths that are available but mutually exclusive with the darker ones due to shared gate control. (b) Parallel architecture. Similarly, the red and blue gate arrays represent independent conveyor belts, with the green gate acting as a horizontal barrier that controls the interaction between two spins located in the conveyors adjacent to the gate. (c) Simulated potential landscapes at different phases of the conveyor cycle for the shelf architecture. (d) Corresponding simulations for the parallel architecture, demonstrating that, under similar conveyor operation, qubits can be brought together at the center barrier and their coupling can be controlled. Other qubits being transported in the conveyors are not affected by this interaction, except for the accumulation of dynamical phase due to position-dependent Zeeman energy, identical to that during regular transport. (e) Connectivity graph for the shelf architecture, where red and blue nodes represent qubits on the lower and upper conveyors, respectively. (f) Connectivity graph for the parallel architecture, with the same color scheme. In all cases, red and blue circles denote qubits located on separate conveyors.

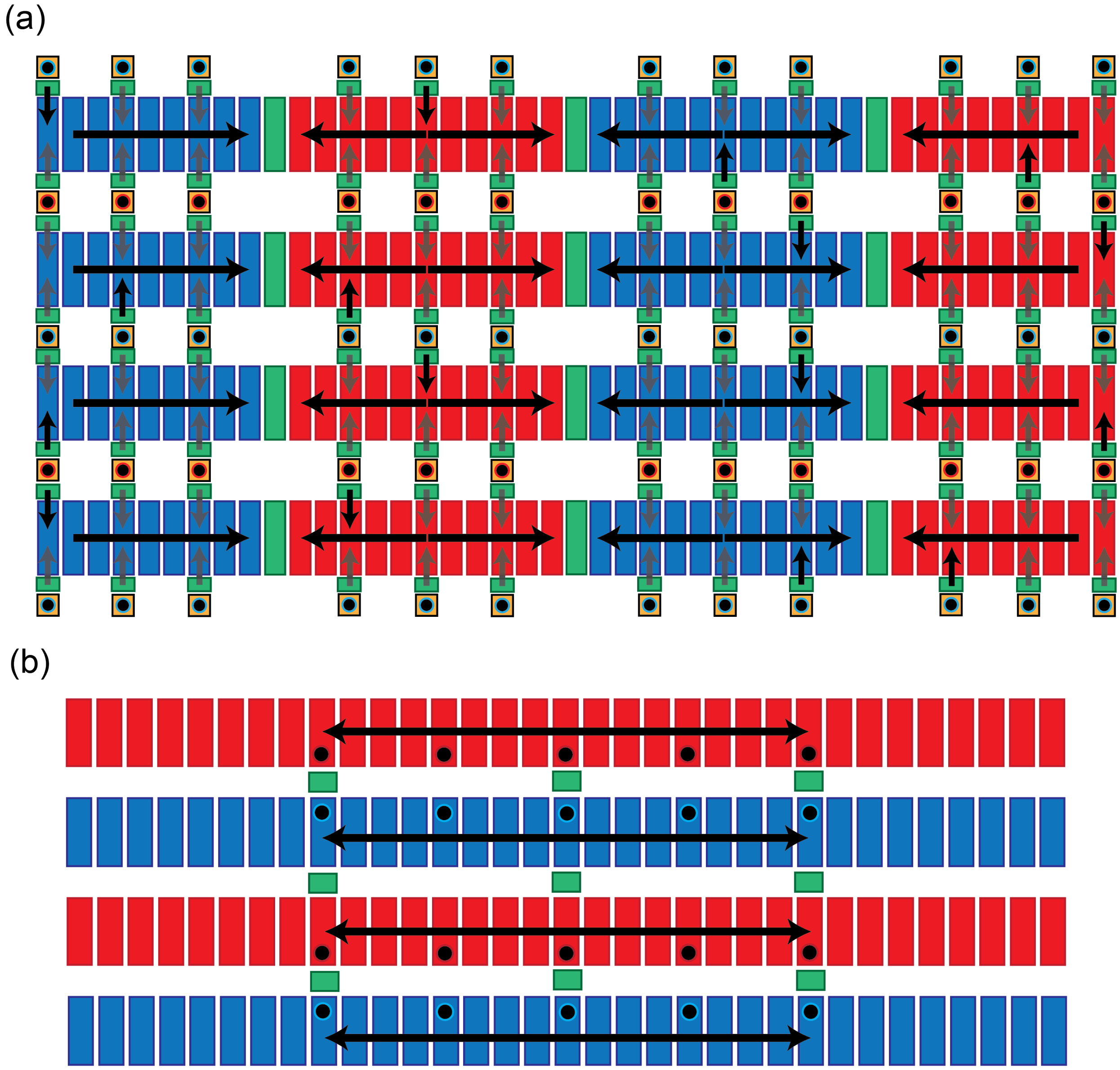

Supplementary Figure 7. **2D integration of mobile spin qubit unit cells** (a) Shelf architecture. The red and blue gate arrays function as independent shared-control conveyor belts, while the green gates denote individually controlled barrier gates. Red and blue circles indicate qubits stored to the side of the conveyors. (b) Parallel architecture. Similarly, the red and blue gate arrays represent independent conveyor belts, with the green gates acting as individually controlled horizontal barriers. The arrows indicate possible shuttling paths, with their transparency illustrating shared-control constraints. Darker arrows show actively occupied paths in this example, while lighter arrows represent alternative paths that are available but mutually exclusive with the darker ones due to shared gate control.

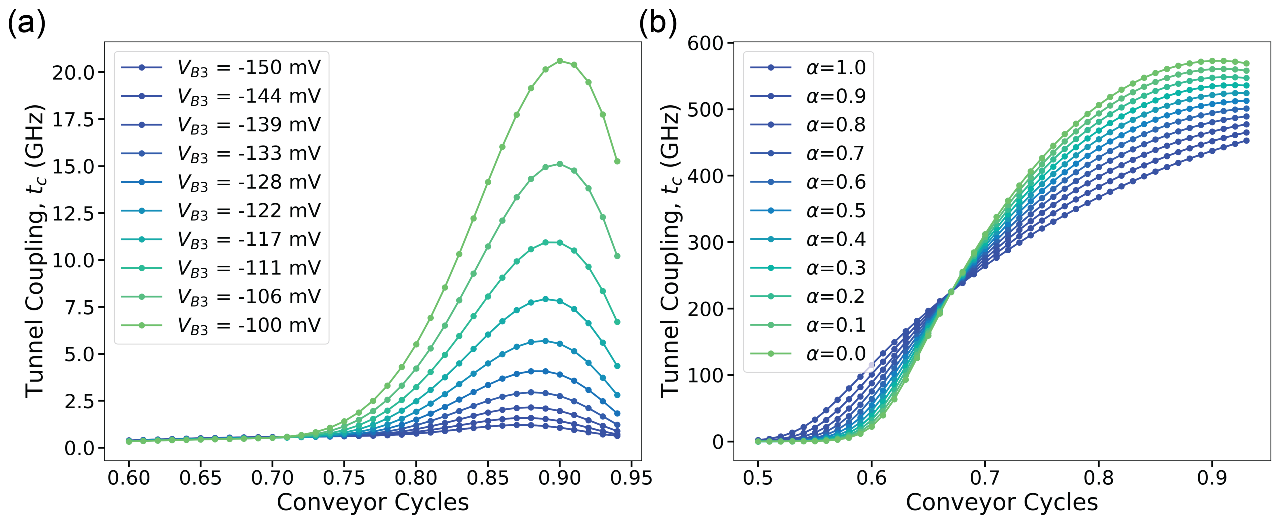

Supplementary Figure 8. **Simulation of tunnel coupling between the two conveyor channels. a) Tunnel coupling dependence on the central barrier voltage for the conditions corresponding to Figure 2c in the main text. Here $A_{\mathbf{1st}}$ and $A_{\mathbf{2nd}}$ are both set to 50 mV an V_{offset} is zero everywhere except for the central barrier, for which the value is indicated in the legend (and labeled V_{B3}. b) The tunnel coupling dependence on the ratio ($A_{\mathbf{2nd}} = \alpha A$, $A_{\mathbf{1st}} = (1-\alpha)A$) with $A = 50$ mV between the first and second tone sine wave pulse amplitude is given for the voltage conditions corresponding to Figure 2e in the main text. Here V_{offset} is zero everywhere, including for the central barrier. A larger value of α results in the dot elongating along the shuttling direction.**

C. Potential simulation methodology

The simulated potential landscape in the main manuscript is modeled by a one-dimensional potential along the transport direction (x). The potential profile along x is expressed in eV, and results from separate contributions due to the voltage on each gate electrode:

$$V_{\text{periodic}}(x,t) = \sum_{i=0}^{N_g-1} V_{\text{gate}}(x - x_i, t)$$

$$V_{\text{gate}}(x-x_i,t) = V_{\text{ref}}(x-x_i) \cdot \Big[A_{\text{1st}} \sin\Big(\frac{\pi i}{2} + 2\pi f t\Big) +$$

$$A_{\text{2nd}} \sin\Big(\frac{\pi i}{4} + \pi f t\Big) + V_{offset}\Big]$$

where N_g is the number of gates and x_i is the position of the i-th gate. A_{1st} and A_{2nd} are the first and second tone sine wave pulse amplitude, respectively, expressed in mV. V_{offset} is a pulsed voltage offset, also in units of mV. $V_{\text{ref}}(x - x_i)$ is the potential in the quantum well as a function of position x resulting from a 1 mV voltage applied to gate i, so $V_{\text{ref}}(x - x_i)$ has units of eV/mV. This potential profile is obtained by solving the Poisson equation at 300 mK with QTCAD [9].

We note that the present simulations do not include static potential disorder (e.g., due to charge traps or interface roughness). In our proof-of-principle experiment, such disorder is typically compensated by applying DC or pulsed voltage offsets to selected gates. Therefore, the simulated potential landscape corresponds to an idealized, disorder-free case.

D. Simulation of the tunnel coupling between moving quantum dots in the two conveyors

To better understand the time evolution of exchange between two mobile spins in the two conveyors, we simulate the tunnel coupling (or orbital energy when the two potential minima have merged) as a function of time while the conveyors travel towards each other.

To calculate the tunnel coupling t_c between the two potential minima, we first simulate the one-dimensional potential landscape at each discrete time step of the conveyor cycle. For each time step, we solve the time-independent Schrödinger equation to find the energy eigenvalues of the ground state (E_0) and the first excited state (E_1). In a double-well potential, the otherwise localized ground states hybridize, resulting in an energy splitting $\Delta E = E_1 - E_0$ which is dependent on both the tunnel coupling t_c and the energy detuning ϵ between the two wells, given by the relation

$$\Delta E = \sqrt{\epsilon^2 + (2t_c)^2}.$$

To isolate t_c, we numerically perform a detuning sweep at each time step. This procedure is necessary because, despite the symmetry of the original geometry and gate biasing, numerical asymmetries introduced by the simulation mesh may prevent the formation of perfectly symmetric and antisymmetric ground and first excited states. The sweep allows us to find the true zero-detuning point empirically. It is achieved by applying a small, varying voltage offset to one of the gates adjacent to the central barrier, which directly controls ϵ. By calculating ΔE across a range of these offsets, we can find the minimum energy splitting, ΔE_{min}. This minimum occurs at zero detuning ($\epsilon \approx 0$), where the energy splitting is directly proportional to the tunnel coupling, i.e., $\Delta E_{\text{min}} = 2t_c$. This procedure is repeated for every time step to map out the evolution of t_c throughout the conveyor cycle.

Figure 8a shows the simulated tunnel coupling as a function of conveyor time, with the simulation parameters chosen to correspond to the experimental conditions in Fig. 2b of the main manuscript. In this protocol, a large negative pulsed voltage offset is applied to the central barrier gate. The purpose of this high potential barrier is to prevent the two potential wells from fully merging. This controlled separation is key to achieving a smoothly varying exchange pulse shape, as it allows the tunnel coupling to be modulated without an abrupt turn-on. The simulation demonstrates that applying a more negative offset voltage systematically suppresses tunneling. This calculated behavior shows good qualitative agreement with the experimental trend observed in Fig. 2b.

Figure 8b illustrates how the shape of the conveyor parameters affects the tunnel coupling profile. The conveyor is composed of two sinusoidal tones with different frequencies. Here, αA represents the amplitude of the second, slower-frequency tone, while the amplitude of the primary tone is set to $(1 - \alpha)A$. By varying α, we change the relative contributions of the two tones,

thereby modifying the shape of the potential wells as they merge. The simulation shows that this waveform engineering provides a powerful method for tailoring the temporal evolution of the tunnel coupling during the interaction.

To better utilize the strongly correlated regime, it may be necessary to dynamically modulate the potential shape during the merging process to minimize dephasing during the ramp. Furthermore, employing a larger gate pitch to create a more elongated potential could allow the electron wavefunctions to merge at a smaller exchange interaction strength, providing even more precise control over the quantum operation than already shown in this first demonstration.

E. Quantitative analysis of error source in CZ gate operation

First, we note that the average Clifford gate fidelity from the reference measurement is 85.18%. This is consistent with the expected fidelity based on the gate decompositions. Using $F_C = 1 - (1.5r_{CZ} + 8.25r_{SQ})$, with an IRB-extracted CZ error rate r_{CZ} = 0.0114 and a joint single-qubit error rate with simultaneous driving r_{SQ} = 0.0146, we estimate F_C = 85.53%.

Then, in order to obtain insight in the main contributions to the CZ infidelity, we analyze the dephasing effects during our CZ gate operation by considering low-frequency fluctuations in the exchange interaction J and individual qubit frequencies, causing random deviations between the actual operation U_{exp} and ideal operation U_{ideal}. We model this as a stochastic unitary U_{exp} dependent on noise parameter x. From the T_2^* measurements at different conveyor periods shown in Figure 2d, we observe T_2^* values ranging from approximately 0.2-4.0 μs during the interaction. We make the following assumption for these fluctuations. The fluctuations are Gaussian distributed with zero mean, stationary , and described by a $1/f$ power spectral density. Furthermore, we assume that the noise only gives rise to an accumulated phase, thus, we can approximate this noise using quasistatic fluctuations. By integrating over the corresponding frequencies, we obtain $\sigma^2 = 2\int_{t_m^{-1}}^{t_e^{-1}} \frac{S_x}{f} df$, where the effective standard deviation of the noise σ is proportional to $1/T_2^*$. For a measurement time t_m and gate operation time t_e, this relationship can be expressed as [10, 11]:

$$\sigma \propto \frac{1}{T_2^*} \propto \sqrt{\ln \frac{0.401}{t_e/t_m}}$$

Here the total time t_e of CZ gate is 58 ns. To estimate the gate infidelity, we compare two measurement time scales: the T_2^* measurement time of 138 s and the IRB measurement time of 5160 s. Using the relationship above, we calculate the effective noise standard deviation σ for the IRB scenario. We calculate the average gate fidelity between the experimental unitary and the ideal CZ gate unitary as:

$$F = \frac{\left\langle \left| \mathrm{tr}\left(U_{\mathrm{ideal}}^{-1} U_{\mathrm{exp}}\right)\right|^2 \right\rangle + d}{d(d+1)}$$

where the expectation value is given by [11, 12]:

$$\left\langle \left| \mathrm{tr}\left(U_{\mathrm{ideal}}^{-1} U_{\mathrm{exp}}\right)\right|^2 \right\rangle = \int_{-\infty}^{\infty} \left| \mathrm{tr}\left(U_{\mathrm{ideal}}^{-1} U_{\mathrm{exp}}(x)\right)\right|^2 \frac{1}{\sqrt{2\pi}\sigma} e^{-\frac{x^2}{2\sigma^2}} dx$$

For the two-qubit system, $d = 4$, the experimental unitary matrix in the computational basis $|\downarrow\downarrow\rangle, |\uparrow\downarrow\rangle, |\downarrow\uparrow\rangle, |\uparrow\uparrow\rangle$ is given by:

$$U_{\mathrm{exp}}(\chi) = \begin{pmatrix} 1 & 0 & 0 & 0 \\ 0 & e^{-2\pi i \int f_1(t,\chi) dt} & 0 & 0 \\ 0 & 0 & e^{-2\pi i \int f_2(t,\chi) dt} & 0 \\ 0 & 0 & 0 & e^{-2\pi i \int (f_2(t,\chi)+f_3(t,\chi)) dt} \end{pmatrix}$$

where $f_1(t,\chi) = f_{\mathrm{Q5,Q2}\downarrow}(t,\chi)$, $f_2(t,\chi) = f_{\mathrm{Q2,Q5}\downarrow}(t,\chi)$, $f_3(t,\chi) = f_{\mathrm{Q5,Q2}\uparrow}(t,\chi)$.

By integrating the qubit frequencies $f_{Qi,Qj}(t,x)$ over time under the conveyor pulse used for CZ operation and the noise amplitude χ, we estimate the total dephasing-induced infidelity during the CZ gate operation to be ~0.22%.

Additionaly, we evaluate the undesired residual SWAP processes during our CZ gate operation through numerical simulations based on a time-dependent Heisenberg Hamiltonian:

$$H(t) = \frac{\Delta E_z}{2}(\sigma_1^z - \sigma_2^z) + J(t)(\sigma_1^x\sigma_2^x + \sigma_1^y\sigma_2^y + \sigma_1^z\sigma_2^z)/4$$

where $\Delta E_z = 83$ MHz is the Zeeman energy difference between the qubits, and $J(t)$ follows the experimental pulse shape with a maximum value of 33 MHz. The first term represents the energy splitting between the $|01\rangle$ and $|10\rangle$ states, while the second term describes the exchange interaction between the spins.

We numerically solve the time-dependent Schrödinger equation using this Hamiltonian to obtain the unitary evolution operator over the complete gate sequence. The fidelity is evaluated by computing the average gate fidelity between the obtained unitary U_{exp} and the ideal operation U_{ideal}. For our experimental parameters, we find a contribution to the infidelity $1 - F = 0.01\%$.

Although the ratio of $\Delta E_z/J \approx 2.5$ at the pulse peak is not sufficiently large to suppress SWAP errors, the actual gate infidelity remains very low. This follows directly from the expected pulse shape of J that follows a smooth curve. The error from the SWAP process is $\sim S(-|\Delta E_z|)^2$, where $S(-|\Delta E_z|)$ is the spectral density of the exchange noise evaluated at the Zeeman energy difference. Remarkably, a linear ramping pulse translates to an almost Gaussian window that strongly suppresses SWAP processes [13].

The total estimated infidelity (0.22% from dephasing and 0.01% from imperfect adiabaticity) remains smaller than the experimentally observed infidelity of approximately 1.14%. The remaining infidelity could possibly be attributed to extremely low-frequency discrete shifts in qubit parameters that might occur on timescales longer than the T_2^* measurement time or thermal effects (heating) during operation that shift qubit frequencies [2] and induce additional high-frequency noise in the device.

F. Characterization of polarization and phase information teleportation

Figure 9a provides a guide for the required feedforward operations based on the Bell measurement outcome and the corresponding parity readout results. Due to the inability to distinguish states when the first parity readout is odd, we focus the analysis on post-selected cases where we detect either the Ψ^+ or Φ^-– Bell states. These cases correspond to applying X and Z gates, respectively, to the teleported spin state.

Figure 9b illustrates that the spin polarization prepared via Rabi oscillations in Q6 is successfully transferred and observable in the state of Q2, as expected. We note that this step in itself only confirms that a classical probability was transferred, and provides only partial evidence for successful quantum state teleportation. The blue and red curves show the oscillations after post-selecting the Bell states Ψ^+ and Φ^-, respectively. In this case, no tomography pulses are applied.

Next, we teleport superposition states prepared with different phases in Q6. This is achieved by applying an $R_x(\pi/2)$ pulse followed by an $R_Z\ (\theta_1)$ rotation on Q6 during state preparation. Post-selecting on the Ψ^+ Bell state, the phase information is transferred to Q2 where we apply an $R_Z(\theta_2)$ rotation followed by another $R_x(\pi/2)$ pulse, which projects the phase information onto the Z-basis measurement. Figure 9c shows the resulting oscillations, which depend on both the $R_Z\ (\theta_1)$ operation applied to Q6 and on the $R_Z\ (\theta_2)$ rotation on Q2. These oscillations confirm that the phase information is preserved and accurately transferred through the teleportation process. Nevertheless, similar to Fig. 9a, this is only partial evidence of successful quantum state teleportation.

G. Quantitative analysis of error sources in quantum state teleportation

We analyze the error sources in our teleportation protocol, beginning with characterizing the entangled Bell states through quantum state tomography. Fig. 9(a) shows the reconstructed density matrix for the entangled state between mobile qubit Q2 and qubit Q5, generated via shuttling-based CZ operations, while Fig. 9(b) presents the density matrix of the entangled state between qubit Q5 and qubit Q6, prepared using a local CZ gate in the static dots. From these measurements, we extract Bell state fidelities of $90.2 \pm 1.3\%$ for the Q2–Q5 pair and $83.9 \pm 1.6\%$ for the Q5–Q6 pair by fitting the phase of the reconstructed density matrices and calculating their overlap with the closest theoretical Bell states, obtained through quantum state tomography with maximum likelihood estimation. The uncertainties are the standard deviation obtained through bootstrap resampling. Using the relation

$$F_{\text{tele}} = \frac{1 + 2F_{\text{Bell}}}{3},$$

we estimate that the error contributed by Bell state preparation for Q2–Q5 is $\sim 6.5 \pm 1.3\%$.

The Q5–Q6 pair, while characterized with a Bell state fidelity of 83.9%, serves a different purpose in the protocol. These qubits are used for the Bell measurement basis transformation rather than directly contributing to the teleportation channel. The fidelity of operations on this pair affects the measurement process rather than directly transferring to teleportation infidelity in a straightforward manner. The error analysis needs to consider the distinct roles of different qubit pairs in the teleportation protocol. While the Q2-Q5 Bell state directly impacts the teleportation channel quality with an estimated error contribution of $6.5 \pm 1.3\%$, the measurement process involving Q5-Q6 has a more complex relationship with the overall protocol fidelity. This complexity was observed experimentally in the teleported Rabi oscillations (Figure 9c), where the amplitude varied significantly depending on the post-selected Bell state. When the $|\Phi^-\rangle$ state was post-selected, accurately estimating the process tomography

(a)

Initial state	Ψ^+	Φ^-	Φ^+	Ψ^-
State after basis change	$\|00\rangle$	$\|11\rangle$	$\|01\rangle$	$\|10\rangle$
Parity readout outcomes	even + even	even + odd	odd + odd	odd + odd
Required feed-forward	X	Z	I	ZX

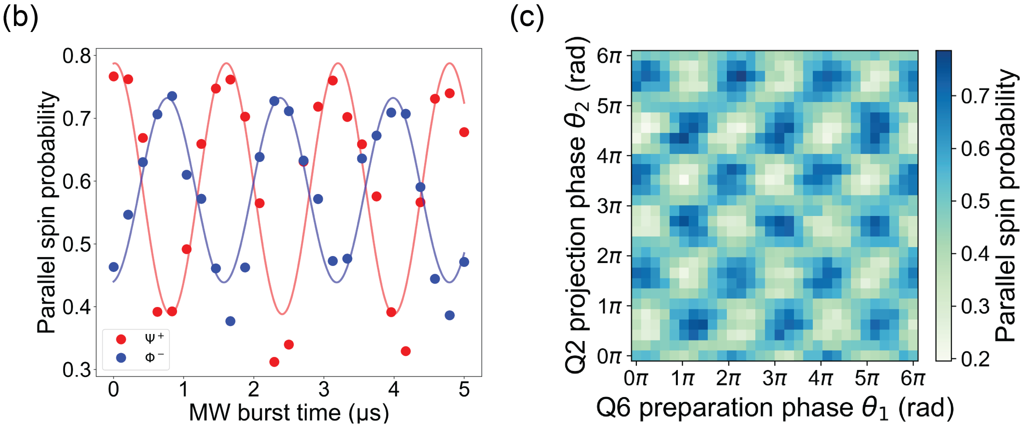

Supplementary Figure 9. Bell measurement and teleportation of either the spin polarization or phase. a) Look-up table relating the input states to the computational basis states they are mapped to by the basis transformation, the parity readout outcomes given the practical constraints of successive parity measurements, and the required feed-forward operations to complete teleportation. b) Parallel spin probability measured on Q1Q2 as a function of the microwave burst time on Q6, showing Rabi oscillations on Q2 after teleportation, post-selected on detecting Ψ^+ (blue) and Φ^-– (red) Bell states. c) Two-dimensional map of the parallel spin probability measured on Q1Q2 after teleporting a superposition input state, post-selected on detecting the Ψ^+ Bell state.

matrix (PTM) through quantum process tomography (QPT) proved challenging, indicating that the error mechanisms manifest differently across the four Bell measurement outcomes.

We evaluate the average fidelity of the parity readout between Q5 and Q6 directly from the Rabi oscillation data. The return probability for the $|1\rangle$ state is measured to be $P_1 \approx 0.9799$, while the minimum probability (corresponding to the $|0\rangle$ state) is $P_0 \approx 1 - 0.0088 \approx 0.9912$. The average readout fidelity is then $F = \frac{P_1+P_0}{2} \approx 0.9856$, resulting in an average error per parity readout of $\varepsilon \approx 0.0144$. Since two parity readouts contribute to the overall fidelity, the combined error from the parity readouts is roughly 2.9%, though this estimation reflects the overall SPAM error, and the actual readout error is expected to be lower.

We note that the parity PSB readout error of Q1-Q2 used for teleportation verification is not part of the teleportation process itself, so we removed it using the following matrix:

$$\begin{pmatrix} 0.951 & 0.125 \\ 0.049 & 0.875 \end{pmatrix} .$$

The elements represent the probability of measuring each state given the prepared state: correctly measuring $|0\rangle$ when $|0\rangle$ was prepared (0.951), incorrectly measuring $|0\rangle$ when $|1\rangle$ was prepared (0.125), incorrectly measuring $|1\rangle$ when $|0\rangle$ was prepared (0.049), and correctly measuring $|1\rangle$ when $|1\rangle$ was prepared (0.875). To remove the readout error from our data, we processed the probabilities as follows. If $P_{|1\rangle}$ is the probability of measuring $|1\rangle$ in our data, we calculated:

$$\begin{pmatrix} P^{\text{corrected}}_{|0\rangle} \\ P^{\text{corrected}}_{|1\rangle} \end{pmatrix} = \begin{pmatrix} 0.951 & 0.125 \\ 0.049 & 0.875 \end{pmatrix}^{-1} \begin{pmatrix} 1 - P_{|1\rangle} \\ P_{|1\rangle} \end{pmatrix} .$$

Overall, while multiple error sources contribute to the protocol performance, the key areas for improvement include optimizing the Bell state preparation fidelity and enhancing the Bell measurement process in the static dots. Particularly, improving the local CZ gate performance between Q5 and Q6 would benefit the measurement basis transformation, which in turn would enhance the overall teleportation fidelity.

Fig. 11 shows the average gate fidelities for four successive runs of the teleportation protocol without recalibrations in between. Each runs takes approximately 7 minutes to complete. As the measurements are repeated, parameter drift causes a gradual decrease in fidelity. Since the Bell state tomographies were performed after the final (4th) measurement, the estimated errors closely represent the conditions at the end of the experiment, in good agreement with the observed results.

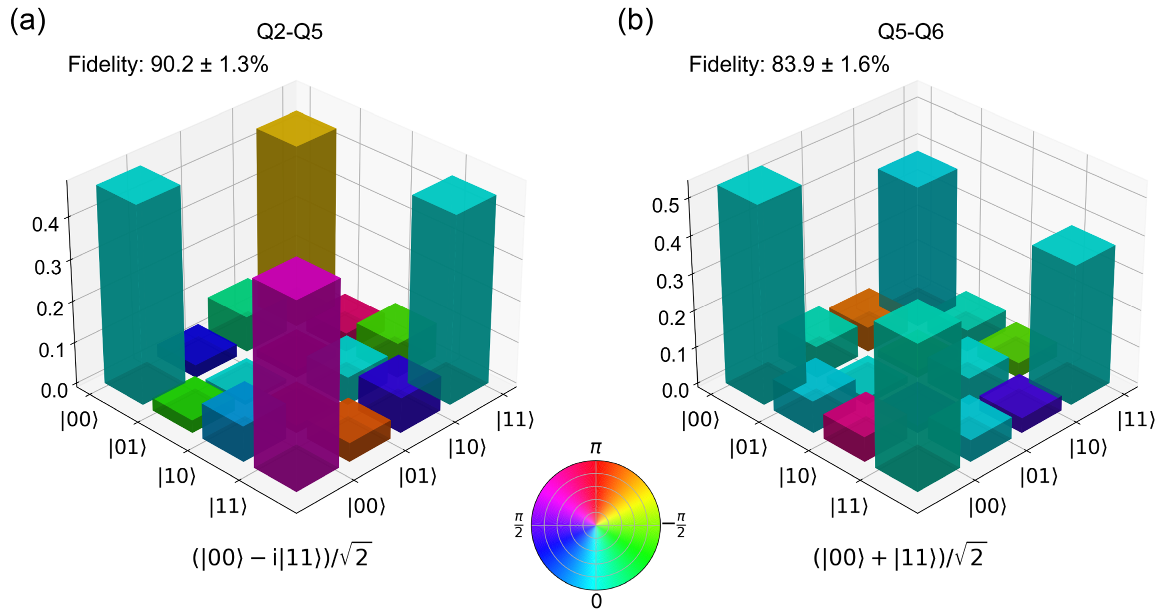

Supplementary Figure 10. **State tomography of entangled spin pairs used for teleportation.** a) Reconstructed density matrix of the entangled state between mobile qubit Q2 and qubit Q5, prepared using shuttling-based controlled-phase operations. The basis states are ordered as $|00\rangle$, $|01\rangle$, $|10\rangle$, $|11\rangle$. We extract a Bell state fidelity of $90.2 \pm 1.3\%$ for this Q2–Q5 pair by fitting the phase of the reconstructed density matrix and calculating its overlap with the closest theoretical Bell state. b) Reconstructed density matrix of the entangled state between qubits Q5 and Q6, used as a resource for teleportation, with an extracted Bell state fidelity of $83.9 \pm 1.6\%$. Both density matrices were obtained through quantum state tomography with maximum likelihood estimation. Both real and imaginary components are represented by the bar heights, and the phase indicated by the color scale in radians.

[1] Degli Esposti, D. *et al.* Low disorder and high valley splitting in silicon. *npj Quantum Information* **10**, 1–9 (2024).
[2] Undseth, B. *et al.* Hotter is easier: Unexpected temperature dependence of spin qubit frequencies. *Physical Review X* **13**, 041015 (2023).

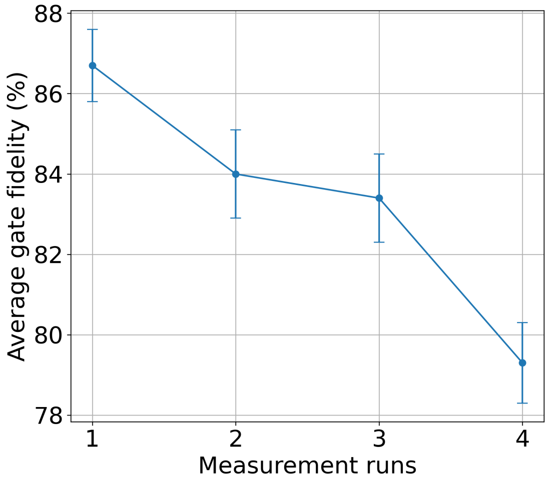

Supplementary Figure 11. **Average gate fidelities obtained from quantum process tomography (QPT) of the quantum teleportation protocol across four measurement runs.** The horizontal axis indicates the measurement run, while the vertical axis shows the average gate fidelity (%). Error bars represent one standard deviation obtained from bootstrap resampling in each run. As time progresses, the initially tuned parameters drift, leading to a decrease in fidelity. Each runs takes approximately 7 minutes to complete.

[3] Mills, A. *et al.* High-fidelity state preparation, quantum control, and readout of an isotopically enriched silicon spin qubit. *Physical Review Applied* **18**, 064028 (2022).

[4] Lawrie, W. I. L. *et al.* Quantum dot arrays in silicon and germanium. *Applied Physics Letters* **116**, 080501 (2020).

[5] Degli Esposti, D. *et al.* Wafer-scale low-disorder 2DEG in ^{28}Si/SiGe without an epitaxial Si cap. *Applied Physics Letters* **120**, 184003 (2022).

[6] Langrock, V. *et al.* Blueprint of a scalable spin qubit shuttle device for coherent mid-range qubit transfer in disordered Si/SiGe/SiO_2. *PRX Quantum* **4**, 020305 (2023).

[7] Siegel, A. *et al.* Snakes on a plane: mobile, low dimensional logical qubits on a 2d surface (2025). arXiv:2501.02120.

[8] Ohira, R. *et al.* Trapping an atomic ion using time-division multiplexed digital-to-analog converters. *arXiv:2508.04093* (2025).

[9] Nanoacademic Technologies. QTCAD: Quantum transport cad tool. https://nanoacademic.com/solutions/qtcad/ (2023). Accessed: 2026-01-16.

[10] Cywiński, L., Lutchyn, R. M., Nave, C. P. & Das Sarma, S. How to enhance dephasing time in superconducting qubits. *Phys. Rev. B* **77**, 174509 (2008).

[11] Wang, C.-A. *et al.* Operating semiconductor quantum processors with hopping spins. *Science* **385**, 447–452 (2024).

[12] van Dijk, J. *et al.* Impact of classical control electronics on qubit fidelity. *Phys. Rev. Appl.* **12**, 044054 (2019).

[13] Rimbach-Russ, M., Philips, S. G. J., Xue, X. & Vandersypen, L. M. K. Simple framework for systematic high-fidelity gate operations. *Quantum Science and Technology* **8**, 045025 (2023).
